# Models of endometriosis and their utility in studying progression to ovarian clear cell carcinoma

**DOI:** 10.1002/path.4657

**Published:** 2015-12-23

**Authors:** Claire M King, Cynthia Barbara, Andrew Prentice, James D Brenton, D Stephen Charnock‐Jones

**Affiliations:** ^1^ Department of Obstetrics and GynaecologyUniversity of Cambridge, The Rosie HospitalCambridgeUK; ^2^ Functional Genomics of Ovarian Cancer LaboratoryCR‐UK Cambridge Institute, University of Cambridge, Li Ka Shing CentreRobinson WayCambridgeUK; ^3^ National Institute for Health ResearchCambridge Comprehensive Biomedical Research CentreCambridgeUK

**Keywords:** endometriosis, clear cell ovarian cancer, endometrioid ovarian cancer, menstruation, animal model

## Abstract

Endometriosis is a common benign gynaecological condition affecting at least 10% of women of childbearing age and is characterized by pain – frequently debilitating. Although the exact prevalence is unknown, the economic burden is substantial (∼$50 billion a year in the USA alone) and it is associated with considerable morbidity. The development of endometriosis is inextricably linked to the process of menstruation and thus the models that best recapitulate the human disease are in menstruating non‐human primates. However, the use of these animals is ethically challenging and very expensive. A variety of models in laboratory animals have been developed and the most recent are based on generating menstrual‐like endometrial tissue that can be transferred to a recipient animal. These models are genetically manipulable and facilitate precise mechanistic studies. In addition, these models can be used to study malignant transformation in epithelial ovarian carcinoma. Epidemiological and molecular evidence indicates that endometriosis is the most plausible precursor of both clear cell and endometrioid ovarian cancer (OCCA and OEA, respectively). While this progression is rare, understanding the underlying mechanisms of transformation may offer new strategies for prevention and therapy. Our ability to pursue this is highly dependent on improved animal models but the current transgenic models, which genetically modify the ovarian surface epithelium and oviduct, are poor models of ectopic endometrial tissue. In this review we describe the various models of endometriosis and discuss how they may be applicable to developing our mechanistic understanding of OCCA and OEA. © 2015 The Authors. *The Journal of Pathology* published by John Wiley & Sons Ltd on behalf of Pathological Society of Great Britain and Ireland.

## Introduction

Endometriosis is defined as the presence of endometrial tissue (glands and stroma) outside the uterine cavity; it is found primarily in the pelvis and can involve all organs but particularly the ovaries and pelvic peritoneum. It is a common condition affecting an unknown number of women of reproductive age. Typical symptoms include pain and subfertility, and the complexity and severity of the pain can present major management challenges 1, 2. Dysmenorrhoea affects up to 62% of women with endometriosis, and is incapacitating in up to 28% 3.

Definitive diagnosis requires laparoscopy or laparotomy and histological confirmation, but there is often a protracted time to diagnosis (mean of 8.0 and 11.7 years in the UK and USA, respectively) 4, due to the complexity of the presenting symptoms. The annual economic costs associated with delays in diagnosis are substantial and were estimated at approximately $50 billion in the USA 5. Management of pain symptoms often combines analgesia, hormonal therapies such as hormonal contraceptives, progestagens (either systemic or more commonly local delivery of levonorgestrel to the uterus), or gonadotropin‐releasing hormone (GnRH) analogues. Surgical treatments include ablation or excision of endometriotic lesions or ovarian cystectomy and occasionally hysterectomy and bilateral salpingo‐oophorectomy. The treatment of associated subfertility is challenging and may be surgical or by the use of assisted reproduction such as IVF. However, surgery carries risks of complications which may themselves compromise fertility. Finally, adhesiolysis and treatment of lesions carries a risk of injury to all pelvic structures 6. These treatment modalities vary in their effectiveness in relieving the pain and increasing pregnancy rates, and also in their side‐effect profiles 7.

The concentration of a range of inflammatory mediators – cytokines, growth factors, eicosanoids, and angiogenic factors – is altered in the peritoneal fluid of women with endometriosis 8, 9, 10. Disturbance of several of the immunomodulators has been implicated in the endometriosis‐associated infertility 11. Here, we review the relationship between endometriosis and clear cell and endometrioid ovarian cancers and the experimental models that may be used to investigate the mechanisms underlying disease progression.

### The origin of endometriosis

At present, there is no definitive answer for the origin of endometriosis, but the most widely accepted theory is that it arises from endometrium that has disseminated to ectopic sites, most commonly by retrograde menstruation 12, 13, 14. Thus, menstrual tissue passes through the fallopian tube and into the peritoneum, where it attaches, invades, and grows into endometriotic lesions. There is a genetic component to endometriosis risk and a genome‐wide association study has identified candidate risk alleles 15.

Endometriosis has high prevalence, significant morbidity, and considerable economic impact. The wide range of treatments used in clinical practice underscores our limited understanding of the pathogenesis of endometriosis and approaches that exploit new models and analytical techniques could have high utility. Furthermore, although endometriosis itself is a benign disease, several epidemiological studies have identified it as a risk factor for some types of malignancies, most notably ovarian cancer, and particularly ovarian clear cell adenocarcinoma (OCCA) or ovarian endometrioid adenocarcinoma (OEA). Models which allow the underlying mechanisms to be elucidated offer the prospect of patient stratification and over the longer term, new therapies.

### Endometriosis and ovarian cancer

Epithelial ovarian cancers (EOCs) are not a single disease but rather a group of diseases with distinct morphological and molecular features, all of which may affect the ovary. The principal subtypes of EOCs are high‐grade serous carcinoma, low‐grade serous carcinoma, mucinous carcinoma, clear cell adenocarcinoma (OCCA), and endometrioid adenocarcinoma (OEA). These tumours likely have different cells of origin, distinct histopathological features, and exhibit different molecular profiles. However, all subtypes of EOCs are treated by surgical debulking and platinum‐based chemotherapy. OCCA and OEA have high rates of primary resistance to platinum‐based chemotherapy which contributes to their poor prognosis when presenting with late stage or reoccurrence.

### Epidemiology

Endometriosis is now recognized as a likely precursor of some subtypes of epithelial ovarian cancer. As early as 1925, Sampson made the observation that cancer developed in ectopic endometrial tissue 16. Since then, multiple epidemiological studies have investigated the association between endometriosis and ovarian cancer. Two large studies were carried out using the Swedish Inpatient Register (NSIR) and National Swedish Cancer Registry. In the first 17, the authors studied all patients discharged from hospital with a diagnosis of endometriosis between 1969 and 1983 – a total of 20 686 patients. Among patients with well‐documented endometriosis, there was an increased risk of ovarian cancer (SIR, defined as ‘the ratio of the observed numbers of cancer to those expected’, 1.9; 95% CI 1.3–2.8) and the risk was particularly elevated in subjects with a long‐standing history of ovarian endometriosis (SIR 4.2; 95% CI 2.0–7.7). The second study also used the NSIR to identify a large cohort of women diagnosed with endometriosis between 1969 and 2000 18. When 25 430 women with ovarian endometriosis were analysed, the risk of ovarian cancer was increased, with an SIR of 1.77 (95% CI 1.38–2.24), and this peaked 3–4 years after hospitalization to an SIR of 2.64 (95% CI 1.2–5).

A pooled analysis of 13 case–control studies (23 144 women) was used to examine the consistency and degree of association between endometriosis and the different subtypes of ovarian cancer 19. Self‐reported endometriosis was consistently found to be associated with an increased risk of invasive epithelial ovarian cancer after taking into account confounding factors (including study site, ethnic origin, oral contraceptive pill use, and parity). Risk was strongly associated with OCCA and OEA subtype (OCCA odds ratio 3.05, 95% CI 2.43–3.84, *p* < 0 · 0001; OEA odds ratio 2.04, 95% CI 1.67–2.48, *p* < 0.0001; and low‐grade serous odds ratio 2.11, 95% CI 1.39–3.20, *p* < 0.0001). However, the history of endometriosis in these case–control studies was self‐reported and this may predispose to recall bias. While it is presumed that cancer cases may be more likely to report a history of endometriosis than controls, it is likely that this bias is random and may not account for the difference in association between the different subtypes of ovarian cancer.

### Molecular evidence for the relatedness of endometriosis to OCCA and OEA

The consistent finding of increased risk in the above epidemiological studies supports a two‐ to three‐fold increase in the risk of OCCA and/or OEA in patients with endometriosis. However, it is plausible that a common characteristic, such dysregulation of cytokine production or steroid metabolism within the peritoneal environment, predisposes to both conditions. However, there are several lines of molecular evidence that suggest that OCCA and OEA arise directly from endometriotic lesions. The first published studies used information from loss of heterozygosity (LOH) surveys in OCCA and OEA cases to test whether identical changes were also present in adjacent endometriosis. In a study of 19 OEAs, 22 OCCAs, and 23 solitary endometrial cysts, LOH at 10q23.3 (the gene locus of *PTEN*) occurred in 42.1% (8 of 19) OEAs, 27.3% (6 of 22) OCCAs, and 56.5% (13 of 23) solitary endometrial cysts. Importantly, in five OEAs and seven OCCAs with *synchronous* endometriosis, three cases in each group showed identical LOH events in both tumour and endometriosis; one case in each group had LOH in the tumour only. The remaining endometriosis cases (one OEA and three OCCAs) did not show LOH events. None of the endometriotic lesions showed LOH events that were not also present in the co‐existent tumours 20. These data suggest that loss of *PTEN* function by LOH or mutation is an early event in the development of endometriosis‐related cancers of the ovary.

Similar findings were demonstrated using 82 microsatellite markers to detect LOH in ten OEA and six OCCA cases with synchronous endometriosis. A total of 63 LOH events were identified in the tumours, with 22 found in the matched endometriosis tissue, and in each case, the same allele was lost. LOH was not noted in control endometriosis‐only cases 21.

More recently, specific mutations and copy number abnormalities have been demonstrated in tumour and co‐existent endometriosis. Wiegand *et al* sequenced the entire exome of 18 OCCA samples and one OCCA cell line, and the *ARID1A* gene in an additional 210 ovarian cancers 22. *ARID1A* was mutated in 46% of OCCAs and 30% of OEAs, with 17 of the samples having two somatic mutations each. No *ARID1A* mutations were found in high‐grade serous (HGS) specimens. Two of the patients who had OCCA samples with *ARID1A* mutations also had contiguous atypical endometriosis (ie with epithelial cells showing nuclear enlargement, crowding, slight hyperchromasia, and possible chromocentres/nucleoli and/or architectural abnormalities). One of these patients had a truncating mutation and the other had two somatic mutations predicting loss of BAF250a protein (encoded by *ARID1A*). These mutations were found in the contiguous atypical endometriosis but not in distant endometriosis, further supporting local transformation of endometriosis tissue. Immunohistochemical staining for BAF250a was also lost in both OCCA and the contiguous atypical endometriosis but not in distant endometriosis 22. Taken together, these data strongly support a model for early mutation of *ARID1A* during malignant transformation of endometriosis.

These results were confirmed using immunohistochemistry for BAF250a 23 in an independent series of matched OCCA and endometriosis tissues. In that study, the authors also sequenced exons 9 and 20 of *PIK3CA* and found synchronous mutations in 17 of 42 (40%) of these tumours, most of which (71%) were also *ARID1A*‐deficient 23. This suggests that *PIK3CA* mutations also occur at a very early stage in the development of OCCA, before the appearance of the atypical precancerous lesions.

The *MET* proto‐oncogene is frequently overexpressed in OCCA and is frequently amplified. Copy number alterations and MET protein levels were studied in 13 tumours and precursor lesions [including 11 endometriosis and two clear cell adenofibromas (CCAFs)]. *MET* copy number gain and overexpression were demonstrated in the atypical precursor lesions. Non‐atypical endometriosis and benign CCAFs did not demonstrate *MET* gain 24. These data suggest that *MET* gain is an early event in tumourigenesis in a subset of OCCAs and that it is a driver mutation.

To further identify the pattern of somatic mutations in OCCA and investigate whether there was a clonal relationship between benign gynaecological lesions, including endometriosis, and OCCA, whole‐genome sequencing of seven OCCAs was performed and targeted sequencing of other co‐existent adjacent or metastatic tumours, endometriosis, and other benign conditions 25. Most of the mutations found in the index tumour were also found in the other co‐existent cancer or borderline tumours in the same patient. There were clusters of mutations present in both OCCA and at least one focus of atypical or non‐atypical endometriosis from the same patient. No somatic mutations were found in normal endometrium or non‐endometriosis benign lesions. Thus, atypical endometriosis shared nearly all of the coding somatic mutations that were detectable in the co‐existent tumour and it is highly likely that these mutations are ancestral events in the evolution of the clear cell tumour. These findings support a model in which different subtypes of endometriosis have differing malignant potential – however, the morphological diagnosis of atypical endometriosis is difficult and these data may also suggest a local field effect for mutational change.

The studies described above, considered collectively, make a compelling case for endometriosis being the precursor lesion of OCCA and OEA – although this progression is rare. The major clinical challenge is now to determine which individuals are at higher risk of the rare event of transformation of endometriosis to OCCA or OEA. However, our understanding of the molecular basis of this progression is far from complete. Effective patient screening and therapeutic intervention will only be possible once we have a fuller picture of the molecular pathways involved. Consequently, in the absence of any effective screening for any ovarian cancer type, current guidance is that no action be taken to monitor this potential progression in women with endometriosis 6. Such investigations are not possible in human subjects, so it is necessary to use animal models. However, these models need to be carefully selected and their limitations recognized. The establishment, development, and rare transformation of endometriosis take place in a complex environment and the selected model should replicate this as closely as possible while allowing specific manipulation and appropriate analysis. The importance of the tissue microenvironment and the selective genetic modification at defined times has been well described in cancer models 26. Such refined approaches should inform the design of studies of endometriosis. Here, we review the animal models used in the study of endometriosis and explore their suitability for improving our understanding of endometriosis and whether they represent appropriate models to investigate the rare progression of endometriosis to ovarian cancer.

## Animal models of endometriosis

For any system to be a useful model of a human disease, it needs to recapitulate the typical features of that condition but to be manipulable in a way that humans are not. Thus, there may be a naturally occurring veterinary condition that parallels the human disease; if so, this could be used to investigate the molecular mechanisms underlying the disease. If this is not the case, then a more interventional approach is needed. The simplest and the most commonly accepted pathological mechanism underlying endometriosis is the retrograde menstruation proposed by Sampson 27. All the models of endometriosis assume this mechanism and rely on transferring endometrium to a site outside the uterine cavity.

### Spontaneous endometriosis in primates

Unlike other model organisms, some primates menstruate and develop spontaneous endometriosis and this is very similar to the human condition. For example, rhesus monkeys (*Macaca mulatta*) develop endometriosis that “anatomically and clinically, appears to be identical to the human disease” 28. The frequency in a captive breeding colony was ∼1%, although in a more recent study of animals aged ≥ 10 years the prevalence was ∼31% 29. In baboons (*Papio anubis*), the frequency is broadly similar – 11% or 32% in animals held in captivity for less than 1 year or greater than 2 years, respectively. This difference may be due to an increase in the number of menstrual cycles (due to a reduction in the number of pregnancies) 30.

### Artificial induction of endometriosis in primates

Spontaneous endometriosis in primates can take several years to develop. However, to study the onset and progression of the disease, the variability and length of time required for development of endometriosis are a major limitation. This has been partially alleviated by the development of methods that artificially induce endometriosis in primates. In 1953, Scott *et al* diverted the normal menstrual flow by repositioning the cervix and clamping the descending uterine vessels 31. The ten rhesus monkeys used in this study were then monitored a year later, after which five had viable endometrial stroma and glands, smooth and striated muscle, and fibrous tissue in the area to which the diversion was made. Similar results were obtained in baboons in which the volume of retrograde menstrual flow was increased by surgical occlusion of the cervix 32.

### Induction of endometriosis by transplantation

Endometriosis is characterized by endometrial tissue being ‘in the wrong place’. Hence, a reasonable approach to making an experimental model is to collect endometrium and transplant to an ectopic site.

Iatrogenic endometriosis as a complication of surgery was seen to be so common that Ridley in 1968 wrote “this fact is now growing so commonplace that it is accepted and usually not reported in the literature” 33. Thus, it is not surprising that the obvious approach of autologous transplantation in primates was used to generate an animal model of endometriosis 34. Surgically excised fragments of endometrium were transplanted into a number of sites in the peritoneum of rhesus monkeys. Six of the seven monkeys had viable grafts up to 522 days after transplantation. Other studies have used minced or enzymatically digested endometrium in cynomolgus monkeys (*Macaca fascicularis* **)**. After 3 weeks, 76% of the subjects had endometriosis 35. When endometrial fragments were collected during the menstrual phase and introduced into the peritoneum of baboons by injection, endometriosis developed in a very similar manner to the spontaneous disease 36. This model showed that endometriosis could be caused by material introduced using less invasive methods and was thereby similar to humans in that the fragments seed wherever they land. However, as the material was collected by curettage, it contained tissue from layers of the endometrium that would not be found in menstrual material. A similar model, but using a pipelle (endometrial suction curette) to collect tissue at the time of menstruation, was used in the identification of ERα, MMP‐7, VEGF, and aromatase in endometriotic lesions 37, 38.

The effects of the presence of endometriosis on the eutopic endometrium have been studied in humans 39, and similar questions have been addressed using the baboon model. Endometriosis was induced and uterine endometrium sampled in the mid‐secretory phase 1, 3, 6–7, 10–12, and 15–16 months after endometriosis induction. Transcript profiling was carried out and showed that during the early stages of the disease, there was an oestrogenic dominant phenotype in the eutopic endometrium. As the condition progressed, the endometrium became progesterone‐resistant. In addition, several signalling pathways, including the ERK/MAPK and PI3/AKT, were dysregulated and expression of genes such as *KRAS*, *FOS*, and *NODAL* was altered 40. Using the same approach to induce endometriosis led to increased Cyr61 (an angiogenic protein) in the eutopic endometrium 41.

In many ways, primate studies are attractive because these animals are closely related to humans, anatomically and physiologically, and spontaneously develop the disease, suggesting that the peritoneal environment is similar. These studies exemplify the usefulness of the baboon as a model animal for the study of endometriosis – the endocrine environment is similar to that in humans and can be well controlled; the induction of endometriosis is uniform and under tight temporal control; it is possible to visualize the lesions and collect samples over a prolonged time; and, lastly, the animal cycles naturally. This is counterbalanced by more complex handling and housing requirements; thus, experimental work with primates is costly and ethically challenging.

### Rodent and rabbit models

Laboratory animals do not menstruate and never develop spontaneous endometriosis. However, hamsters, rabbits, and particularly rats and mice are widely used as scientific models for medical research, due to their short generation times and their ease of physiological and of particularly genetic manipulation. Rabbit and rodent models of endometriosis have been established based on transplanting endometrium or uterine fragments, either from the same species (homologous models) or from humans (heterologous models), to ectopic sites.

A rabbit model of endometriosis was generated to study the effects of endometriosis on fertility 42. Endometrium was transplanted from one horn of the uterus into the peritoneum. The concentration of prostaglandin in the peritoneal fluid increased and the pregnancy rate dropped 25%. Transplanted material was still present 12 weeks later, and had become cystic and similar to the lesions seen in humans 43. Such studies suggested that the presence of endometrium in the peritoneum caused changes in the local environment, which could explain the reduced fertility. This rabbit model was useful for identifying changes in the peritoneal environment and has the advantage that the ectopic tissue grows without supplementary oestrogen and the host ovaries and immune system are intact.

### Homologous rodent models

Both rat and mouse models have been generated by autotransplantation of fragments of the uterus to the mesentery 44, 45. This tissue developed into fluid‐filled cysts which grew for approximately 2 months, before remaining stable for at least 10 months (in rats). In both species, epithelial and stromal cells were present. However, in this model, both the endometrium and the myometrium were transplanted, and so the resulting cysts were made up of both endometrial and myometrial tissue. Given that endometriosis by its very nature is endometrium‐derived, this model does not represent an ideal model of the disease. When the endometrium was scraped from the myometrium and ‘flushed’ into the peritoneal cavity, no lesions developed 44.

A model in which only the endometrium is transplanted has been developed. The endometrium was collected from ovariectomized and oestrogen‐treated mice. It was minced and injected into the peritoneum of syngeneic recipient animals 46. The recipients were also treated with exogenous oestradiol and the resulting lesions were allowed to develop for ∼3 weeks. Endometriosis is an oestrogen‐dependent disease; indeed, as already described, many of the therapeutic strategies rely on reducing circulating oestradiol or opposing its action. This model uses high doses of oestradiol to stimulate endometrial growth before collection and then similar doses to stimulate lesion growth in the recipient animals. The authors recognize that this may be one of the reasons why they were able to generate lesions when previous attempts without oestrogen stimulation failed. This strong oestrogen drive may limit the usefulness of this model and women with endometriosis do not have elevated oestradiol levels.

An enhancement of this model which improved the quantitative assessment of lesion growth made use of donor mice in which green fluorescent protein (GFP) was ubiquitously expressed 47. As the donor cells are all genetically tagged and can be readily detected by fluorescent microscopy, the location and the intermixing of donor and recipient cells can be observed. These authors also demonstrated that the lesions grew poorly in the absence of exogenous oestradiol 47.

While it is possible to image GFP in the internal organs of a mouse 48 (and hence endometriotic lesions), the sensitivity is relatively low. Bioluminescent imaging using luciferase‐expressing cells has greater sensitivity and allows non‐invasive monitoring of lesion development. Transgenic mice with the human ubiquitin C promoter coupled to firefly luciferase have been used as the donors in the endometriosis model described above 45, 49. The uterus of a *UbC‐Luc* ^+/+^ mouse was collected and the horns were opened to allow numerous 2‐mm tissue samples to be punched out. Four of these were then sutured into separate peritoneal locations in albino recipient mice. The luciferase‐derived signal level was measured 2 weeks post‐surgery and showed a clear signal in the location of suture sites. The mice were culled 2 weeks later and the lesions examined. All showed similarity to human endometriosis lesions, with evidence of angiogenesis. This approach is a significant advance as lesion growth can be serially monitored non‐invasively for up to 35 days 49. The origin of infiltrating cells can be tracked in this model using genetically tagged donor (with GFP, for example) or recipient animals. This model is well suited to monitor lesion response to drugs that inhibit new vessel formation 50. Nonetheless, it has the limitations of the transplantation models described above, due to the presence of both the endometrium and the myometrium.

### Spontaneous mouse endometriosis

In 2005, the first mouse model in which endometriosis developed without any form of transplant was described 51. Activating *K‐ras* in *lox*P‐Stop‐*lox*P‐*K‐ras^G12D/+^* mice (*LSL‐K‐ras* ^G12D/+^) by injecting Cre‐adenovirus into the ovarian bursa resulted in the development of endometriotic‐like lesions (endometrial glands but no stroma) in the ovarian surface epithelium in all the mice, and endometriotic lesions with both glands and stroma in the peritoneum in 47% of the animals. When similarly transduced ovaries were transplanted, the endometriotic‐like lesions were limited to the ovarian surface epithelium. This suggests that the endometriotic‐like lesions on the ovary in this mouse model most likely arose from the ovarian surface epithelium itself. However, the peritoneal lesions probably arose from uterotubal cells in which K‐ras was activated along the needle track. Injection of AdCre virus directly into the peritoneal cavity of the *LSL‐K‐ras* ^G12D/+^ mice did not lead to the development of any endometriotic‐like lesions, suggesting (in mice at least) that endometriosis does not arise through metaplastic transformation of the peritoneum 51. These authors exploited their novel methodology and introduced additional mutations. Injection of AdCre virus into the ovarian bursa of *LSL‐K‐ras* ^G12D/+^;*Pten* ^loxP/loxP^ mice leads to signs of invasive ovarian endometrioid adenocarcinoma between 7 and 12 weeks later. However, the unintended transduction of tubular cells (which was clearly demonstrated in this study) may in fact be an important characteristic of this method of delivering the Cre. While the method provides clear information about the function of the genes manipulated, conclusions about the precise cell of origin need to be drawn cautiously. Nonetheless, the presence of the *K‐ras* mutation in this model overcame the need for additional hormone stimulation and provides a useful model to study the histomorphology and biological behaviour of endometriosis.

### ‘Menstrual’ rodent models

Endometriosis is a condition in which normal menstrual material persists in an abnormal environment. Thus, an animal model of this condition would ideally mimic the processes that allow this material to adhere within the peritoneum and develop into a lesion. Indeed, this was the basis of the initial models in primates. Clearly, rodent models have many attractions but they do not menstruate naturally.

A mouse model has been developed to mimic the endometrial breakdown seen in human menstruation. In this model, ovariectomized mice are treated with oestradiol and progesterone and the endometrium is stimulated to decidualize by injecting a small amount of oil directly into the uterine lumen. Examination 48 h after cessation of the hormone injections (ie progesterone withdrawal, as occurs at the end of the menstrual cycle) revealed blood vessels filled with swollen erythrocytes, apoptotic changes, and invasion of leukocytes. By 79 h post‐withdrawal, blood cells and degenerating decidual cells had been shed into the uterine lumen 52. This model is suitable for exploring the processes that take place in humans following luteal regression; however, one of the limitations of this model is the variation in the endometrial response.

This model has been refined: C56J/Bl6 ovariectomized mice were exposed to a programme of oestrogen injections to mimic the levels seen in the human cycle. However, progesterone was delivered via an implant inserted prior to the injection of oil into the uterus. The removal of the implant 49 h after the decidualizing stimulus allowed a more dramatic drop in serum progesterone levels, more closely representing the human cycle. Mice were sacrificed and the uterine response was examined 16, 24, 36, and 48 h post‐progesterone withdrawal. After 16 h, breakdown of the endometrium was observed; by 24 h, the decidual zone had separated from the rest of the endometrium. Tissue debris was fully cleared by 36 h after progesterone withdrawal and by 48 h, the endometrium was restored to its pre‐decidual state 53. A similar study with additional molecular analysis has also been carried out 54.

These two studies form the basis of related but distinct models of endometriosis 55, 56. In both cases, mouse endometrium is decidualized by hormone pretreatment and an interuterine injection of oil. Progesterone treatment is withdrawn and 2.5–4 days later, ‘menstrual’ endometrium is collected and transferred to a recipient animal. The two models differ in the duration and strength of the progesterone‐stimulated decidual growth: Cheng *et al* do not give progesterone after the injection of the oil into the uterine lumen 55. In contrast, Greaves *et al* leave a progesterone implant in place for 4 days after the interuterine oil injection 56.

The work of Dinulescu *et al* (described above) showed that activation of *K‐ras* was sufficient to generate an endometriosis‐like lesion in the peritoneum of mice 51. These authors suggested the possibility of “the peritoneal endometriosis having a uterine or tubal origin” in the *LSL‐K‐ras* ^G12D/+^ mice. Thus, a small number of cells in which the LSL‐K‐ras^G12D/+^ had been activated were sufficient to lead to endometriosis.

**Figure 1:**
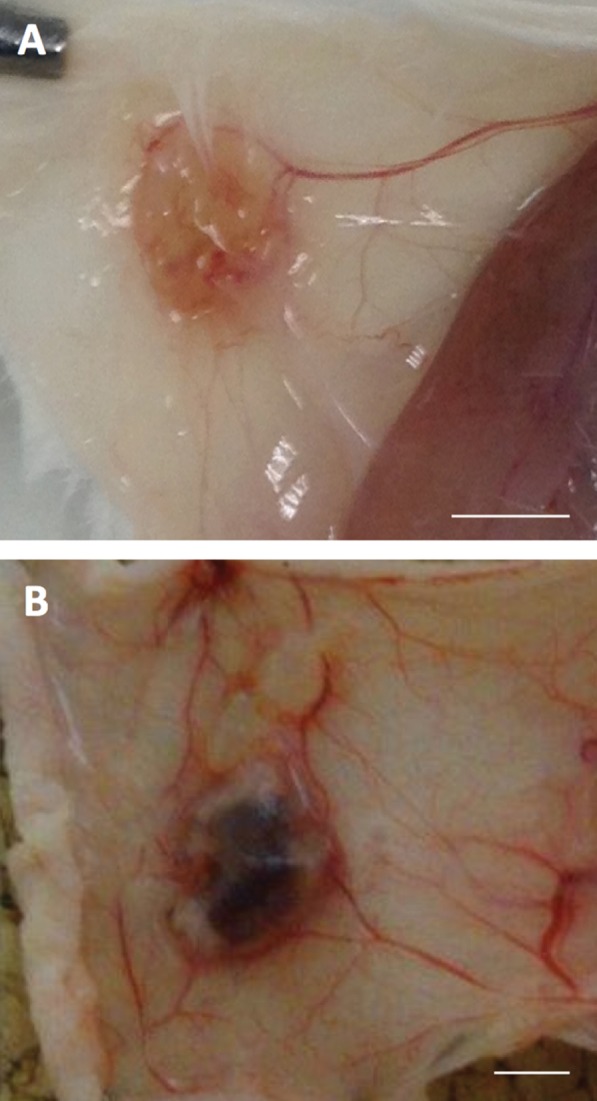
Examples of lesions generated using the models described as ‘menstrual’ rodent models and heterologous rodent models in the main text. (A) Subcutaneous endometriotic lesion in a C57Bl/6 Tyr^−/−^ mouse following transplantation from LSL‐K‐ras ^G12V/+^ /Ah‐Cre ^+/+^ /ROSA26R‐LacZ ^+/+^ mice following the method of Cheng et al 55. (B) Subcutaneous endometriotic lesion in a xenograft model in a Balb/c nu/nu mouse. Scale bar = 2 mm. The work to produce this figure was carried out in accordance with the UK Home Office Animals (Scientific Procedures) Act 1986.

Cheng *et al* used a *LSL‐K‐ras* ^G12V/+^ */Ah‐Cre* ^+/+^ */ROSA26R‐LacZ* ^+/+^ mouse in which Cre was induced in the uterus by β‐naphthoflavone dissolved in the oil used as the decidualizing stimulus 55. The Cre then locally activated the mutated (active) *K‐ras* and the *LacZ* reporter. After progesterone withdrawal, this endometrium was transferred to a subcutaneous pocket in wild‐type intact animals. No exogenous steroids were administered to the recipient animals but lesion growth could be inhibited by treatment with an oestrogen receptor antagonist (fulvestrant, ICI182780). In the absence of the genetic modification, the ‘menstrual’ endometrium did not form lesions. Somewhat surprisingly, only a small proportion of the cells in the endometriotic lesions were LacZ‐positive, suggesting that Cre‐mediated recombination did not occur in all the endometrial cells, but that this low level of *K‐ras* activation was sufficient for lesion establishment 55. These authors did not describe the fate of the engineered menstrual tissue if it was injected into the peritoneal cavity. Nonetheless, lesion growth was robust and accompanied by the growth of surrounding blood vessels that supplied the lesion, as illustrated in Figure 1A.

The extended progesterone treatment during decidualization used by Greaves *et al* is more efficient and yields a greater mass of ‘menstrual’ material for transplant. However, a significant difference in this model is that the recipient animal is primed with and continues to receive oestradiol; thus there is a strong oestrogenic drive which promotes endometriotic lesion growth 56. Despite this limitation, this model has the advantage that it is simpler than that of Cheng *et al* as there is no need for complex animal breeding. This is a considerable advantage as it facilitates the inclusion of other genes of interest (reporter or functional). Indeed, this approach has been effectively used to show that both host‐ and shed tissue‐derived macrophages are present in the growing lesions 56.

### Heterologous rodent models

An alternative to auto‐ or syngeneic‐transplant is to use human endometrium and immunodeficient mice as recipients. Collectively, these have the advantage that human tissue is used, so drugs or antibodies with species‐specific effects can be used. However, they have the disadvantage that the host immune system is severely compromised. Given that endometriosis is recognized as an inflammatory condition, this is a significant weakness in these models 1.

Multiple studies have described the transplantation of proliferative, secretory, and menstrual phase human endometrium into athymic nude mice 57, 58, 59, 60, 61, 62. Tissue has been placed in or injected into the peritoneal cavity 59, 60, 62, or transplanted subcutaneously 58. In some of the studies, the mice were ovariectomized and treated (or not) with oestradiol 57, and in others, the ovaries remained intact 60. Given what is known about human endometriosis, it is not surprising that in animals treated with exogenous oestradiol, the lesions had a higher proportion of proliferating cells 61.

Studies using this model have shown that angiogenesis is required for lesion growth, murine endothelial cells grow into the lesion, and that it is VEGF‐A derived from the human tissue that drives this 60, 63; indeed, numerous blood vessels can been seen around the developing lesion shown in Figure 1B. Another useful characteristic of this model is that host and donor cells can be distinguished. An additional advantage to that obtained by using fluorescently labelled donor cells (as described earlier 47) is that specific transcripts derived from the host or donor can also be identified 64.

These studies broadly agree on the importance of oestradiol, angiogenesis, and invasion for lesion growth. However, the models are not well suited to investigating the underlying mechanisms. For example, there is considerable heterogeneity in the tissue source – human endometrium or endometriotic lesions 65, and a variety of additional different immunodeficient mouse strains have been used (NOD‐SCID 61 and RAG‐2/γ(c)KO 65). Uniform genetic modification of the human tissue fragments is challenging and while the mice can be modified by breeding in the alleles of interest, immunodeficient mice tend not to breed very well. In an interesting approach to tackle some of these limitations, Masuda *et al* prepared a single‐cell suspension of the glandular and stromal portions of human endometrium and transplanted this under the kidney capsule of ovariectomized NOD/SCID/γ_c_^null^ mice. The mice were treated with oestrogen pellets with or without cyclic injections of progesterone. All mice transplanted were found to have endometrium‐like tissue in the kidneys when collected 10 weeks later, compared with only one of the 12 non‐hormone‐treated mice. Mice that received oestrogen and progesterone produced larger cysts than those treated with oestrogen alone. Introduction of a YFP or luciferase gene into the human cells prior to transplantation allowed lesion growth to be followed for 8 weeks using non‐invasive imaging. A cyclic increase and decrease in signal could be detected in the mice when progesterone was given or withdrawn, showing that the cyclic events of the menstrual cycle could be recapitulated in this model 66. Such a model would also be useful in testing potential treatments of endometriosis. However, this model is limited as the recipient is immunocompromised and the site of transplantation is not typical of endometriosis, and so does not aid our understanding of adhesion to the peritoneal surface or the seeding of the ovary.

## Overview of endometriosis models

As summarized above, recent animal models of endometriosis have become more refined and now are able to recapitulate the presumed pathogenesis of the human condition. As might be expected, primate models are the closest to human disease but are ethically difficult and very expensive. Rodent models, which mimic the deposition of menstrual tissue, are now available and have the advantage of being easily genetically manipulable and immunocompetent. However, the need for exogenous oestradiol in rodents may be a limitation – for example, if studying a process that is directly oestrogen‐dependent. However, the complex breeding required to use the model described by Cheng *et al* is a significant disadvantage 55. The principal features of the available models are summarized in Table 1.

**Table 1:** Summary of the main characteristics of the animal models of endometriosis

Model [ref]	Tissue transplanted	Menstrual tissue used	Immunocompetent	Exogenous oestrogen required	Genetic modification needed	Genetically manipulable
Baboon 36	Endometrium	Yes	Yes	No	No	No*
Rabbit 42	Endometrium	No	Yes	No	No	No*
Rat 44	Myometrium and endometrium	No	Yes	Yes	No	Yes
Mouse 45	Myometrium and endometrium	No	Yes	Yes	No	Yes
Mouse 46	Endometrium	No	Yes	Yes	No	Yes
Mouse 51	NA	No	Yes	No	Yes	Yes
Mouse 55	Endometrium	Yes	Yes	No	Yes	Yes
Mouse 56	Endometrium	Yes	Yes	Yes	No	Yes
Mouse 57	Human endometrium	Yes/no†	No	Yes/no‡	Yes	Yes§

* The donor is not currently genetically manipulable and although in principle *ex vivo* viral transduction would be possible, this has not been demonstrated.

† Human endometrium from any phase of the cycle forms lesions in this model.

‡ Exogenous oestradiol is frequently used but is not absolutely required.

§ The donor is not genetically manipulable, although in principle *ex vivo* viral transduction is possible and this has been presented in abstract form (O144) [74].

## Mouse models of epithelial ovarian cancer

It is now understood that ovarian cancer is a heterogeneous disease originating from different epithelial cells in the Müllerian tract, which show marked tropism for spread and growth on the ovary. As early as 1925, Sampson recognized this and said of ovarian endometriotic lesions: “The ovarian implants are usually more active than the latter (peritoneal), thus suggesting that ovarian tissue is the most fertile soil” 16. Thus, any ovarian cancer model used to study the development of the disease should take into account the tissue or cell of origin and the environmental cues that may determine growth at ectopic sites. Recent elegant studies have begun to shed light on the mechanisms which may explain why the ovary is the favoured site for tumour deposition and invasion 67. Labelled ovarian cancer‐initiating cells (OTICs) were injected into the uterine horns of superovulated mice. As early as 5 days later, fluorescent signal could be detected around the ovary by *in vivo* imaging. Histological examination showed labelled cells close to site of the corpus luteum and disrupted epithelium. After 10 days, the cancer cells had invaded the ovarian stroma, leaving an intact ovarian epithelium. While this experiment used OTICs derived from high‐grade serous carcinoma, it demonstrates that these cells are attracted to the ovary. This is likely to be the case for the other types of epithelial ovarian cancers whose cells of origin are different but which lodge in the ‘fertile soil’ of the ovary.

The work of Dinulescu *et al* described above was a significant advance in the development of mouse models of ovarian disease because of the innovative targeting of genetic modifications to a specific site 51. The method has been used to modify other genes implicated in ovarian cancer. For example, *Arid1a* loss and *Pik3ca* activation after intrabursal injection of AdCre in *Arid1a^fl/fl^*(GT)*Rosa26Pik3ca* ^*H1047R^ mice leads to rapid development of ovarian cancer with OCCA‐like histology 68. Approximately half of the mice had peritoneal metastasis, often in the contralateral control (uninjected) ovary, again pointing to the propensity of tumour cells to seed in the ovary 67. This study replicates the two molecular defects that are known to co‐exist in human OCCA and thus can be used to study preventative or therapeutic options. However, a weakness of this study is the fact that OCCA is induced from OSE – or possibly uterotubual cells as found by Dinulescu *et al* 51. If the tumour in fact arose in the OSE, then the model is not applicable to the study of the role of other Müllerian epithelia or other possible precursors, such as endometriosis in the development of OCCA. Similarly, the ablation of *Arid1a* and *Pten* (a phosphatase that inhibits the Pik3ca signalling cascade) using intrabursal AdCre in *Arid1a^fl/fl^;Pten^fl/fl^* animals led to the development of endometrioid ovarian tumours 69.

The Wnt/β‐catenin pathway has been implicated in OEA by mutational profiling studies and functionally tested using models similar to those outlined above 70. *Apc* ^loxP/loxP^ *Pten* ^loxP/loxP^ mice only develop tumours (with the histological features characteristic of OEA) after intrabursal AdCre. The relationship of these pathways to other pathways implicated in ovarian cancer can elegantly be studied using enhancements of this model: for example, the role of *Tp53* (which is infrequently mutated in OCAA but nearly ubiquitous in HGS) 71.

These mouse models replicate many but not all of the features of human OEAs or OCCA, but can now be extended to study in more detail the genetic alterations required for tumour development and progression. This will improve the use of these models to develop new therapies and to study treatment response. However, other models are needed to study the progression of endometriosis into OEA. If in the model the cancer arises in the OSE (the predominant target of intrabursal delivered AdCre), then this is probably the wrong cell type. On the other hand, as the mode of delivery of the Cre (intrabursal injection) also targets a modest number of uterotubal cells, the possibility remains that these cells make a contribution to the pathology observed. The principal features of these models are summarised in Table 2.

**Table 2:** Summary of the main characteristics of the animal models of clear cell and endometrioid ovarian cancer

Model [ref]	Immunocompetent	Genetic modification needed	Genetically manipulable	Genes manipulated	Site of origin of tumour in mouse model
Mouse OEA model 51	Yes	Yes	Yes	*Kras*, *Pten*	OEA probably from endometriosis‐like lesions on ovarian surface epithelium (glands but no stroma)
Mouse OCCA model 68	Yes	Yes	Yes	*Arid1a*, *Pik3ca*	OCCA arose from ovarian surface epithelium
Mouse OEA model 69	Yes	Yes	Yes	*Arid1a*, *Pten*	OEA arose from ovarian surface epithelium
Mouse OEA model 70	Yes	No	Yes	*Apc*, *Pten*	OEA arose from ovarian surface epithelium
Mouse OEA model 71	Yes	No	Yes	*Apc*, *Pten*, *Trp53*	OEA arose from ovarian surface epithelium

## Conclusions

When designing and using animal models of human disease, and specifically endometriosis, it is important to replicate the disease as closely as possible by taking into account the presumed tissue or cell of origin. The models of endometriosis all presume that the underlying mechanism depends on dissemination of endometrium by retrograde menstruation 27, and such models have been developed in non‐human primates and more recently in mice. These allow precise cellular and molecular studies of the pathogenesis of endometriosis but challenges remain. In particular, pain is a critical symptom of endometriosis and the ideal model should enable investigation of the complex interaction between the endometriotic tissue, the inflammatory peritoneal environment, locally growing nerve fibres, and the central nervous system. Some of these interactions are now being investigated 72, 73.

**Figure 2:**
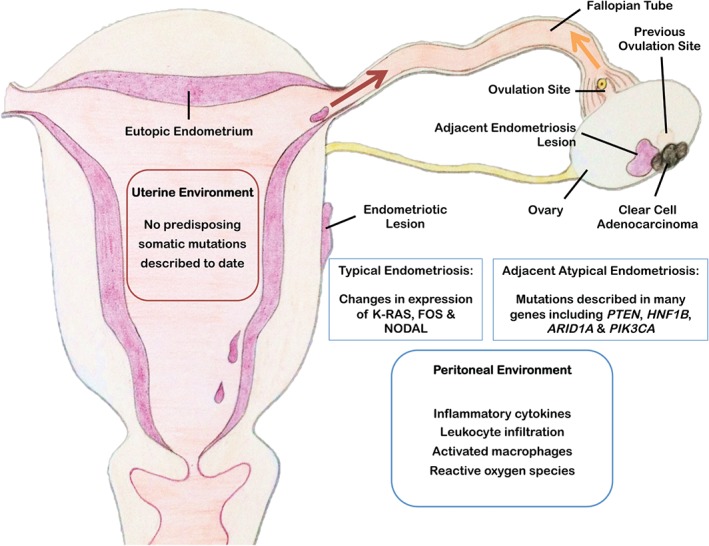
Schematic diagram showing features of endometriosis and postulated mechanisms contributing to the progression of clear cell ovarian cancer. The peritoneal environment in patients with endometriosis is characterized by inflammation 56, 75, 76. Exome sequencing detected somatic mutations in paired eutopic and ectopic endometrium, with a higher burden in ectopic tissue. However, “these mutations occurred in a mutually exclusive manner” 77. Endometriotic lesions and particularly those adjacent to the OCCA carry multiple tumour‐associated somatic mutations 25. The ovary and particularly the ovulation sites are favoured seeding sites for endometriosis and probably tumour cells 67.

The epidemiological and the molecular data reviewed above very strongly suggest that the genesis of OCCA and OEA is not in the ovary – although the ovary is clearly a favoured seeding site. Thus, when developing improved models to study cancer progression, new approaches for targeting endometriosis cells in an appropriate environmental context are now required. The current models very elegantly demonstrate the interplay between genes and do cast light on the processes important for uncontrolled growth in the peritoneal environment. However, they do not fully address the nature of the presumed progression in humans. The nature of somatic changes necessary for the progression from eutopic endometrium to endometriosis, and then (rarely) to ovarian clear cell or endometrioid carcinoma, remains to be defined. As indicated in Figure 2, the peritoneal environment is likely to be a key determinant in this process.

**Figure 3:**
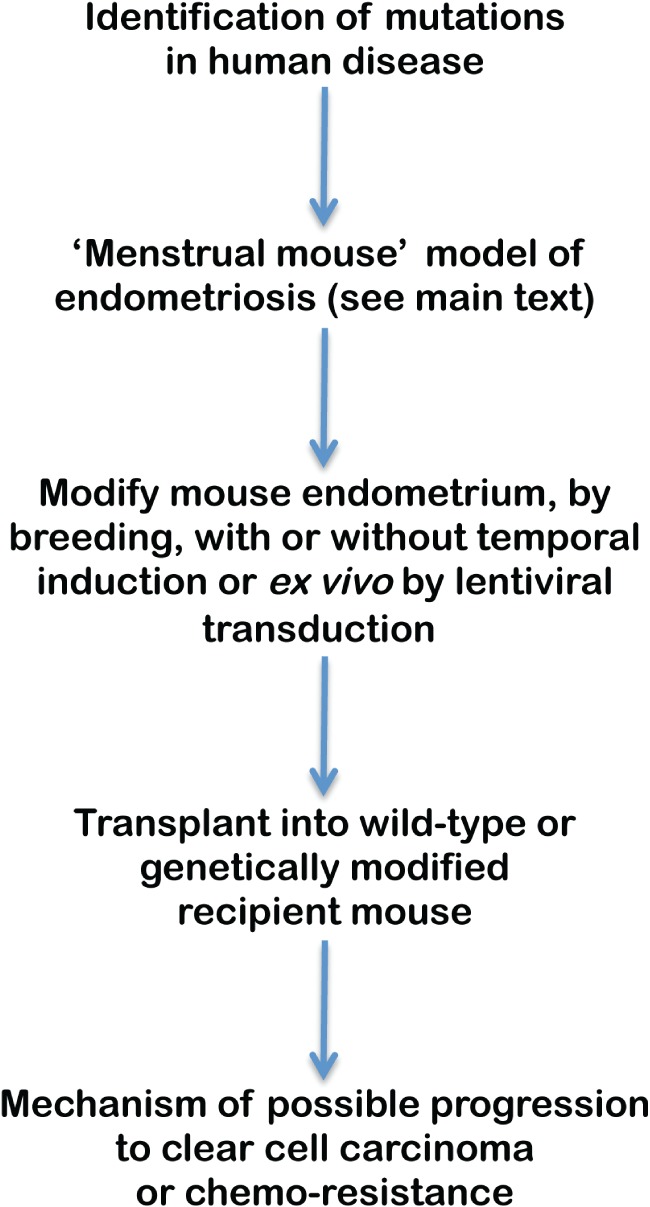
Schematic diagram of how the recently developed models of endometriosis in mice can be applied to the study of progression to clear cell carcinoma.

Over the last 5 years, animal models of endometriosis and those developed to study epithelial ovarian cancer have improved significantly. There is now the prospect of combining genetic modification of murine endometrium with the ‘menstrual mouse’ models of endometriosis to define the role of single or combinations of particular genes in endometriosis and OCCA or OEA. Potential risk alleles identified from human genetic studies can be functionally tested in a model of the presumed precursor lesion. An outline of this approach is shown in Figure 3. Such advances in both fields could lead to new diagnostic and therapeutic strategies and this opportunity should be exploited for the benefit of women.

## Author contribution statement

All authors contributed to the writing of the manuscript and approved the final version.
